# Regulatory and Technical Constraints: An Overview of the Technical Possibilities and Regulatory Limitations of Vehicle Telematic Data

**DOI:** 10.3390/s21103517

**Published:** 2021-05-18

**Authors:** Kevin McDonnell, Finbarr Murphy, Barry Sheehan, Leandro Masello, German Castignani, Cian Ryan

**Affiliations:** 1 Kemmy Business School (KBS), University of Limerick, V94 T9PX Limerick, Ireland; Finbarr.Murphy@ul.ie (F.M.); barry.sheehan@ul.ie (B.S.); Leandro.Masello@ul.ie (L.M.); Cian.Ryan@ul.ie (C.R.); 2 Motion-S S.A., Avenue des Bains 4, Mondorf-les-Bains, L-5610 Luxembourg, Luxembourg; german.castignani@motion-s.com; 3 Faculty of Science, Technology and Medicine (FSTM), University of Luxemburg, Avenue de la Fonte 6, Esch-sur-Alzette, L-5610 Luxembourg, Luxembourg

**Keywords:** connected and autonomous vehicles, intelligent transport systems, telematics, regulation

## Abstract

A telematics device is a vehicle instrument that comes preinstalled by the vehicle manufacturer or can be added later. The device records information about driving behavior, including speed, acceleration, and turning force. When connected to vehicle computers, the device can also provide additional information regarding the mechanical usage and condition of the vehicle. All of this information can be transmitted to a central database via mobile networks. The information provided has led to new services such as Usage Based Insurance (UBI). A range of consultants, industry commentators and academics have produced an abundance of projections on how telematics information will allow the introduction of services from personalized insurance, bespoke entertainment and advertise and vehicle energy optimization, particularly for Electric Vehicles (EVs). In this paper we examine these potential services against a backdrop of nascent regulatory limitations and against the technical capacity of the devices. Using a case study approach, we examine three applications that can use telematics information. We find that the expectations of service providers will be significantly tempered by regulatory and technical hurdles. In our discussion we detail these limitations and suggest a more realistic rollout of ancillary services.

## 1. Introduction

The prevalence of telematic devices is becoming a fixed constant in new vehicles, enabling higher computational capabilities and optimized vehicle performance. Data from telematic devices can contribute to reductions in road accidents and fatalities along with a host of ancillary benefits such as emission reduction, reducing driver distraction, and maintaining or enhancing vehicle value. Inter-vehicle connectivity is also possible through the usage of telematics data, improving traffic flow and vehicle routing. Electric vehicles (EVs) that incorporate the fuel efficiency capabilities afforded by telematic devices will see substantial benefits to battery performance and health. 

The potential of telematic data will be inhibited, however, by both regulatory and technical limitations. The EU has recently published a consultation document to specifically limit third party data access [1]. Future smart systems based on telematics date will be required to adhere to these regulatory limitations. For example, the usage of sensitive geolocation data will be restricted [2]. These regulations will also limit the potential safety and economic benefits afforded by telematic devices. In the context of insurance and infotainment systems, tailored packages for the user will also be limited due to regulation of data. 

Telematic devices are limited by their memory storage, CPU processing, and data transmission capabilities. Improvements in these telematic components are required to handle to the increase in the computational demands of resource heavy applications [3]. Increases in the computational capabilities of the telematic device can contribute additional costs to insurers or users of the device [4,5] The aforementioned technical constraints can further impact the operability of fuel reduction techniques and battery State of Health (SOH) and State of Charge (SOC) systems used in EVs. 

Telematic devices typically monitor acceleration/deceleration, turning force of the vehicle, velocity, and GPS data. Information provided from these devices can be used to assess driver behavior and vehicle performance. Telematic devices are typically installed in vehicles either through black box style installations, embedded systems or through smartphone devices [6,7]. Compared with installed or embedded systems, smartphone devices suffer from sensor precision issues due to their unintended usage as telematic recorders [8]. Telematic devices currently produce 5–15 MB annually, depending on frequency of trips made by the driver [9] These devices are also capable of transmitting the data they record from the vehicle, sending information from the vehicle for usage of high-level applications [10]. Applications that use telematic data can assist in domains such as safety features and services, entertainment services, fuel efficiency, and traffic management systems [11,12].

The potential safety features provided by telematic devices are evident in EU white paper discussions [1,13,14] (European Commission 2016; European Parliament 2018; European Data Protection Board 2020). In the EU 92% of recorded traffic accidents within the last decade are due to driver error [14]. Advanced Driver Assistance Systems (ADAS) can mitigate driver error, as limitations in human perception can be reduced through these automated features. To reduce driver error and improve overall road safety, the EU is introducing regulation requiring the installation of black box recorders, ADAS systems (in particular: intelligent speed assistance, driver drowsiness, attention warning, and advanced driver distraction warning) and tyre pressure monitoring [15].

Safety features using telematics data range from vehicle warning systems to real time traffic and cooperative driving techniques. ADAS enabled vehicles can reduce driver error and improve safety by monitoring driver behaviors and environmental conditions [16]. Connected safety features using telematics data can propagate relevant vehicle metrics to a neighborhood of vehicles [17,18]. Telematics data can be used to alert drivers of nearby vehicle movement intentions and other potential dangers in connected collision warning systems [19].

In-vehicle infotainment describes a supply of services which provide a driver with a range of multi-media, navigation, or networking capabilities. As the transition from manual to autonomous driving progresses, infotainment services will become inclusive to the driving experience. Infotainment services will be provided in a similar fashion to mobile or Internet Service Provider (ISP) packages [20]. Services or applications will be either embedded, tethered, or integrated into the connected vehicle [21]. Drivers can expect a multitude of entertainment services which include games, music streaming videos, weather, navigation, etc. [22]. Third parties can also take advantage of direct in-vehicle communication with the driver and provide geolocation-based advertising [23].

Vehicles fitted with telematic devices can introduce a wide range of energy efficiency benefits, either through optimized driving techniques or improved traffic flow. Data broadcasted in a connected network can enable cooperation between vehicles for efficient traffic flow. Vehicles with Eco-driving capabilities can alert drivers with real time driving optimizations such as optimal acceleration and deceleration [24]. Research conducted by Wu et al. has found that energy consumption can be reduced by 26% when alerting the driver to fuel intensive behaviors [25]. Additionally, two separate studies by He et al. and Wadud et al. have shown that features which encourage a vehicle’s optimal operation (minimize harsh braking and acceleration) can see benefits of 35–55% in energy or fuel efficiency costs [26,27]. Energy efficiency can also be achieved through the usage of network systems. Optimal driver behavior and driver feedback systems have been shown to improve the overall fuel efficiency of a vehicle. Recent advances in inter-vehicle communication technology have afforded researchers the ability to further reduce fuel consumption by using these inter-connected methods. Using a telematic device’s GPS capabilities; optimal routes can be generated for vehicles in the area, reducing travel time or traffic congestion [28]. Efficient traffic flow can be achieved by monitoring acceleration or braking habits of other vehicles [27]. The efficiency of these traffic monitoring techniques has been described by Cox et al. in their paper [29], which show potential energy savings between 13 and 20% in vehicles which utilize this technology.

Telematic devices allow for multiple improvements to efficient traffic flow. Information such as GPS and speed can be broadcast and alerted to other vehicles for smart traffic crossings or intersections. Signal Phase and Timing (SpaT) systems is a method of broadcasting speed and GPS data enabling vehicles to adjust speed to arrive timely on a green light [30]. Intersections or traffic lights can be optimized for efficient traffic flow, as vehicles can safely and optimally transverse through without the need for stopping [31,32]. Broadcasting of GPS information can also be used for re-routing traffic to avoid highly congested areas promoting efficient traffic flow [33,34]. Broadcasting information from neighboring motorway lanes will also have the effect of improving traffic flow and efficiency, as vehicles can change or merge into lanes to promote optimal driving conditions [35,36].

EVs require accurate estimations of battery SOC and SOH to ensure the healthy operation of the battery. Telematic devices can be used to assist in the battery monitoring process. Battey SOC can be estimated by a direct estimation approach or by using model-based methods. Direct estimation algorithms such as amber-hour integral method, estimate the battery SOC directly from the battery [37]. The initial SOC needs to be known however to give accurate estimates of the battery’s current charge. Model-based methods such as Electrochemical Models (EMs), Electrochemical Impedance Models (EIMs), and Equivalent Circuit Models (ECMs), model the state equations of the battery to accurately depict the internal SOC and SOH [38,39]. An adaptive or filtering algorithm which can infer the internal state of the battery will be combined with current state models to create a SOC/SOH estimation model [40]. An improvement on the adaptive model approach is to use machine learning algorithms for accurate and precise predictions of battery SOH and SOC [39].

At an important waypoint before widespread adoption, this research offers a practical assessment of the potential of telematic benefits. Existing research on telematic usage focuses on the benefits and applications of telematic data. However, our research will examine these benefits and applications against the aforementioned constraints. In this way, we highlight the limitations posed by over regulation in a European context and the technical constraints on telematic devices and the impacts this may have on connected vehicles. Risk analysis is also extended to third party services, as regulation may hinder the ability for these services to operate in a restricted environment. Research is conducted as a systematic review utilising a case study approach to examine the risks posed by the constraints on data access and usage from telematic devices.

## 2. Technical Limitations

Vehicle-to-everything is a communication system that allows communication from the vehicle to traffic, infrastructure, and the environment [41]. The current network standards for telematic devices using (V2X) communications, are split between dedicated short-range communications (DSRC) and long-term evolution vehicle-to-everything (LTE-V2X) connections [18]. LTE-V2X utilizes a Third Generation Partnership Project (3GPP) mobile connection for both cellular network communication and direct vehicle-to-vehicle communication. However, there are limitations in the latency and bandwidth of both networking technologies [42]. Two separate studies by Lyu et al. and Elsadig and Fadlalla have found that DSRC suffers significantly with latency issues when introduced to numerous data transmissions in an intersection or traffic jam [43,44]. Lyu et al. further describes DSRC as unsuitable for large networks of connected devices, as low latency is a strict requirement for vehicle safety warnings such as collision warning [43]. The alternative V2X communication, LTE-V2X, is considered unreliable in a paper by Alasmary and Zhuang. In this paper, they describe the limitations of V2X due to loss of packets in concurrent transmissions or blocking of signal by objects [45].

Accurately estimating battery SOH and SOC is challenging. SOC estimations methods which directly monitor the battery such as amber-hour integral requires initial knowledge of the battery SOC to operate. However, determining the SOC of a battery in real-time applications is difficult as this will negatively affect the accuracy of the estimation [37]. Model based methods such as EMs, ECMs and EIMS are criticized by Xiong et al. due to their limited ability to accurately estimate the battery SOC [40]. Machine learning based battery SOH measurements are highly dependent on the transmission of high volumes of data. Acquiring real-time training data for these models using a Machine Learning process are time-consuming and costly [39].

The technical limitations of battery performance monitoring systems reduce the accurate readings of SOC and battery health. Common SOC estimation methods such as EMs, ECMs, and EIMs cannot provide a comprehensive description of battery degeneration and subsequently provide inaccurate readings of SOC [40]. The effects of high SOC levels on battery degeneration and the reduction of the lifetime of the battery has been researched by Wikner and Thiringer, further showing the importance of accurate SOC modelling [46].

## 3. Current and Future Legal Constraints

In the EU, regulation will require the mandatory installation of telematic devices on all new vehicles by 2024 [15]. Vehicles that are fitted with telematic devices can collect a vast amount of personal and private information from the user. Current EU regulations that cover data processing and collection within a vehicle are the ePrivacy Directive [47] and General Data Protection Regulation (GDPR) [48]. The ePrivacy directive requires consent from the data controller in the event of requiring access to or storing of data from a device capable of communication.

The International Working Group on Data Protection in Telecommunications (IWGDPT) and the European Commission have both stated the importance of data privacy and user consent in connection to personal driver data [2,13]. The process of requiring consent should also be afforded to passengers and users of rental vehicles [1,2,49]. The user of the vehicle should also have the ability to delete profile information and other personal information relating to them from the vehicle [1,2]. The European Data Protection Board (EDPB) recommends that manufactures should implement a profile management system in the vehicle. This will allow a user to easily agree, reject, or delete specific data types from different vehicle systems.

The processing requirements of data processing and collection from a vehicle will change. The IWGDPT stipulates that the processing of personal data should be prioritized within the vehicle, as to avoid transmitting sensitive information to remote servers [2] (p. 10). The EDBP endorses this requirement, host-data processing should be prioritized over data transmission. In the event host-data processing is not possible, a qualified third party should be selected to process the raw telematic data [1] (p. 16). The aforementioned regulation ensures that a minimal amount of personal data leaves the vehicle.

## 4. Case Studies

The following case studies have been selected to highlight the impact of technical and regulatory limitations of telematics devices. The three case studies represent diverse applications that are most likely to benefit economically and socially from telematic based applications.

### 4.1. Insurance

The European motor insurance industry is a lucrative market worth GBP 100 billion [50]. Digital based insurance is a growing market currently worth over GBP 15 billon [50]. Third party insurance is mandatory and insurers traditionally group driver risk by location, age, occupation etc. Usage Based Insurance (UBI) is a dynamically priced digital insurance model that monitors driving behavior. GPS data that is collected from telematics devices by the insurer is used to predict driver behavior. Risky driving behavior such as speeding, harsh acceleration, de-acceleration, or cornering can result in higher premiums, while safer behavior will result in lower premiums [51,52,53,54]. Insurers thus incentivize safer driving, saving money for the policyholder and provide bespoke insurance. Monitoring driving behavior requires a device capable of recording and transmitting driver behaviors and vehicle status.

There are numerous benefits to UBI insurance schemes for both insurer and policyholder. Aside from a reduction in premium price, policyholders can expect economic benefits (fuel efficiency, and vehicle condition), safety benefits (feedback on driving behavior, and reduced accident risk) and security benefits (recovery of stolen vehicle) [55,56]. For insurers, more accurate pricing and enhanced fraud prevention and detection capabilities are a significant advantage of using UBI schemes [55,57]. Despite this business appetite, there are regulatory and technical constraints that hamper the implementation of this service.

EU regulation stipulates that data protection and privacy are an integral part of any system which processes or records data [1,2,13,14]. Telematic devices are capable of recording sensitive and private information about an individual. Before data processing can occur, policyholders must provide consent allowing data access by third parties [1,48]. Installed devices belong to the insurer and require user consent for installation. Embedded devices however belong to the driver, requiring the insurer to seek consent for data access [1,2].

To ensure full autonomy over data usage, the EU have recommended that processing of data remains in the host vehicle unless transfer is strictly necessary [1]. The purpose of local host data processing is to limit third parties from accessing raw telematic data produced from embedded telematic devices. For insurers, data can either be accessed from the vehicle after host processing has occurred or sent to a third party for processing before usage.

The EU require the installation of embedded telematic devices on all new vehicles by 2024 [15]. Data scoring and aggregation by insurers requires the removal of personal information relating to the policyholder. Geolocation data has been identified by both EDPB and IWGDPT as particularly sensitive and revealing of personal habits [1,2]. Both the EDPB and IWGDPT have recommended restrictions to geolocation data from third parties. Insurers are limited in their capacity to accurately detect fraudulent claims and driver behavior without access to geolocation data.

Regulation can also introduce technical challenges for insurers and Original Equipment Manufacturers (OEMs) of embedded telematic devices. Data transmission to authorities or third parties will be required in the event of a crash, offence, or loss of vehicle (stolen) [1,58]. Data relating to the event will need to be protected and encrypted before transmission occurs. A typical vehicle may produce 10/15 mb of data annually from a telematic device [9], transitioning to a connected vehicle/network will see data production increased up to 25 GB daily [11]. Telematic data required for investigations by insurers can be delayed through either requiring consent or pre-processing before transmission [1]. The storage capabilities of the device introduce a technical requirement/cost as data must not be deleted to assist in the investigatory process.

The technical hurdles to exploit the full advantages afforded by telematic devices exposes a gap between expectations and technical capabilities. The successful delivery of UBI products hinges on the cost of data transmission and telematic devices for both insurers and policyholders. Higher levels of data transmission from a policyholder’s vehicle can incur significant costs to the insurer. Charges will apply to data transmissions on 3GPP architecture (such as LTE-V2X), taking the European data transmission average of GBP 20 per 50 GB, these costs could be significant [59].

Host data processing requires higher specification components to handle the increase in data flow and memory storage. Standard telematic devices will not be suitable for the additional processing requirements. Higher specification components with larger memory and processing capacity comes at a greater cost. For comparison, a standard telematics device is priced at ~GBP 30 while a higher specification device comes at a cost of ~GBP 80 with increased storage and networking capabilities. The higher priced model is built on current processing components, so a device capable of our forecasted storage and networking requirements is expected to incur a higher price. Embedded devices with higher specifications will be synonymous with higher priced/high spec vehicles. Embedded telematic devices also come at the disadvantage to the insurer, as OEMs create the data access points and communication architecture for telematic data retrieval. Data retrieval for the insurer would require permission from the OEM for the usage of the system architecture. In the EU prices for access to vehicle data is charged at GBP 100 [60]. If we assume that an insurer has 20,000 vehicles with telematic enabled policies, the GBP 100 cost per vehicle can contribute an additional GBP 2 million in operational costs for the insurer. However, depending on brand of vehicle each OEM will require a different process for data access and can charge a different price. We suggest that interest in UBI insurance may dissipate as policyholders and insurers are burdened with increasing costs.

The regulatory and operational costs of embedded telematic devices will impact the availability and economic potential of bespoke insurance schemes. Insurers must prepare for restricted data access to avoid legal and compliance issues with the EU. Changes must be made by the insurer to comply with data consent requirements, local host processing and restrictions on data such as geolocation. The legal ramifications for non-cooperation could be significant, incurring a fine of up to 2% of annual revenue [61].

Where host processing is unavailable, the insurer must seek a competent third party to process policyholder data. The tendering process can be costly to the insurer in numerous ways. The process can be time-consuming, lack of competition can affect the contract price and a third party will add compliance issues. The selection of third-party vendors for data processing can price an insurer out of the UBI market. Less competition for UBI insurance schemes can further increase the premium prices for customers.

There is again a gap between the ability of insures to provide UBI and the emerging regulatory landscape. The absence of geolocation data will inhibit the ability to accurately price a policy, detect fraud or calculate the risk profile of a policyholder using telematic data. Driver behavior monitoring and driver feedback systems that require linking geolocation data such as orientation, speed, acceleration, and deceleration to a policyholder will become inoperable due to the inaccessibility of this data. 

### 4.2. Infotainment and Third-Party Services

Infotainment and third-party services will integrate social media, music streaming, and video services with on-board OEM systems. GPS data can be blended with social media, weather apps or music streaming services to provide unique experiences to the user. Shared trip information recommended playlists in certain environments and real time weather updates are all examples of infotainment and third-party services [62]. Third parties will benefit from enhanced advertising capabilities afforded by infotainment services as advertisements can be sent directly to a vehicle [23]. The transition of current vehicles to entertainment hubs is an attractive prospect for third parties and the consumer.

Third party and infotainment services are highly dependent on access to the user/vehicle. To operate within the EU however, service providers are required to ask for user consent before processing can occur [2,13,14]. Third parties must inform users of their intentions for data processing, gaining consent from users to provide their service. Further restrictions apply to the sharing of personal information outside the vehicle [1]. The operability of third-party services are hindered by limited access to data and transmission restrictions.

Geolocation data is regarded as highly sensitive personal information and as a result access is restricted by the EU [1]. Third party services can only access data that is relative to the process. Processing and transmitting data more than necessary is limited [1]. The operability of services hinges on the accessibility of this data. Services which provide real time updates are restricted by their over reliance on freely accessible geolocation data.

To communicate between vehicles effectively, low latency and reliable systems are required. Current limitations are evident in the networking paradigms used for inter-vehicle communication [43]. Services which use vehicle-to-vehicle (V2V) or vehicle-to-infrastructure (V2I) communications may come under strain as the underlying cellular-vehicle-to-everything (C-V2X) communication technology suffers data congestion. Substantial amounts of data processes will inevitability require large storage requirements. The transmission of telematic data coupled with third party services will increase the average data production rate by 25GB [11].

Third party and infotainment services can enrich the driving experience. However, the gap between the potential range of service and regulatory and technical limitations is significant. Services which require geolocation data will suffer due to data restrictions. In some legislative environments, services will not be offered due to these restrictions.

Multiple users of a vehicle require their own personal privacy settings. Shared vehicle or rental vehicle user privacy is further a problem due to the non-uniform privacy requirements. Requiring consent for data access becomes tedious for multiple users of a vehicle. The EDPB have recommended a centralized service for a user’s data privacy preferences to simplify the consent requirement process. However, users or passengers might select to forgo services if the burden of agreeing to multiple terms and conditions is onerous.

Third parties which intend on relating user behavior with products for advertising will need to explicitly state their intentions for data usage. Subsequently, users are less likely to agree to the terms of service if numerous consent requests are made. Location specific advertisements (location of a user in relation to a business) will not have detailed access to a user’s/vehicle’s position.

### 4.3. Energy Reduction Schemes

Greenhouse gas (GHG) reduction is a global challenge. In the EU passenger vehicles contribute 60.6% of the total CO_2_ emissions from road transportation [63]. Telematic devices can assist in promoting efficient driver behavior, thereby increasing fuel efficiency, and reducing emissions. Reductions in emissions is also possible through optimal vehicle routing. GPS and speed information can be broadcasted to a neighborhood of vehicles, diverting traffic from congested areas or for efficient arrival at intersections. EVs can also benefit from the usage of telematic devices as they provide accurate recordings of the current vehicle state.

The technical limitations of battery performance monitoring systems reduce the accurate readings of SOC and battery health. Common SOC estimation methods such as EMs, ECMs, and EIMs cannot provide a comprehensive description of battery degeneration and subsequently provide inaccurate readings of SOC [40]. High SOC levels also contribute to battery degeneration, reducing the lifetime of the battery [46]. The battery is considered at the end-of-life when the charging state is reduced by 20% [64]. Inaccurate readings of the battery and a high state of charge can contribute to 52% reduction in battery lifetime [46]. The average lifetime of a battery is 10 years [46,64], so inaccurate readings of SOC could reduce the battery lifetime to 5 years.

Stringent regulation on data access can have an adverse effect on the optimal fuel efficiency recommendations that will be present in EVs. Methods that model driving behavior for fuel efficiency optimization require access to a driver’s telematic data [65]. Access to raw telematic data will not be freely available, as regulatory requirements for host vehicle processing or transmitting data to a third party is required [1]. 

Battery health monitoring is a key factor in efficiently charging an EV and prolonging the battery lifetime. If the battery health degenerates significantly, the range that the EV can travel will be reduced. The cost of replacement of the battery within an EV will be significant for the driver, typically the battery is valued at 25–30% of the price of the vehicle [66]. The combination of reduced range and high battery replacement costs may contribute to poor market acceptance of EVs.

Battery performance and fuel optimization methods that provide customized behavioral analysis of driving behavior will also be limited by the restricted access to telematic data. Access to personal data will require explicit definitions of how the data is intended to be used and requires consent before processing. The user has the right to deny access to their telematic data or in the case of data transmission, can terminate the process. Data transmission from the vehicle will also be subject to the processing restrictions determined by the EDBP. Models which provide real-time improvements through the transmission of telematic data will become inefficient due to the limitations of data access.

## 5. Discussion

There is significant optimism about the ability of telematics to deliver new services, reduce energy, and improve driver safety. The benefits of telematic devices will become more apparent, as widespread acceptance of these devices in bespoke insurance products, third-party applications, electric, and connected vehicles grows. Based on a case study approach, we have identified the technical and regulatory limitations that will affect the growth of telematic devices. Technical limitations in the memory, processing, and transmission capabilities of the telematic device will introduce limitations in the operations of energy reduction schemes and third-party services. Similarly, existing, and nascent regulatory hurdles will further disrupt the operability of these services. Regulation that restricts access to telematic data and the requirement for host data processing will contribute heavily to these regulatory overheads. A summary of these technical and regulatory limitations can be found in Table 1.

### 5.1. Insurance

In our research, we have described the limitations and restrictions that will affect the introduction of bespoke insurance products. Within the EU, legislation on the requirement of embedded telematic devices in new vehicles will come into effect by 2024. Insurers will not automatically have access to telematic devices and processing and transmitting substantial amounts of telematic data will be costly, in some cases as much as GBP 46.27 per 50 GB. Restricted access to geolocation data will additionally limit the capabilities of accurately detecting fraud and driver behaviors. The restriction of access to data and the requirement of host processing introduces additional operational costs to providing bespoke insurance products. These costs can be significant for some insurers, and they may be forced to either leave the UBI market or amalgamate services.

Insurers will need to seek alternative procedures to accurately detect fraudulent behaviors and offer bespoke insurance products effectively. Procedures such as privacy-by-design [67] or utilising blockchain-based data privacy technologies [68,69] can assist in privacy preservation during the data collection process. Insurers are likely to consolidate and collaborate with other insurers to lobby legislators, to seek additional data processing privileges. Geolocation data access needs to be addressed as it is imperative to model driving behavior and detecting fraud. Popular methods, such as k-anonymity or location obfuscation, can be employed to mask a policyholder’s location, preserving their right to anonymity [70,71]. These methods can give an insurer a general idea of location for fraud detection, driver behavior analysis and real-time tailored risk classification depending on the area, (e.g., city driving compared to driving in rural areas). Although, changing the underlying data processing and collection procedures may cause initial significant infrastructure and financial cost, the reward of increased data access may alleviate these overheads. 

Embedded telematic devices differ by manufacturing standards and requirements. The non-standardization of these devices may lead to compatibility issues or obsolescence of the device, causing issues with delivery of bespoke insurance products [9]. Insurers will need to communicate with multiple manufacturers to ensure that the networking, storage, and processing capabilities of the embedded telematics devices meets their minimum requirements. Insurers will need to decide collectively what minimum processing and transmission requirements are needed to offer bespoke insurance products. Since third-party vehicle insurance is mandatory, regulation on the minimum requirements for embedded telematic devices should be considered by national governments. Efforts have already been made by OEMs to standardize their communication and data access requirements through Extended Vehicle Data architecture [72]. However, legislators and insurers should be careful as this leaves OEMs with a privileged market position [60]. An alternative solution involving a third party should be considered where data is held, and access is provided by the third party. IBM BlueMIX, Caruso and Otonomo are already examples of these services. Our suggestions for mitigating these regulatory and technical constraints are further detailed and summarized in Table 2.

### 5.2. Third Party Services

The burden of multiple consent requirements for users of third-party services as mentioned in Case Study 4.2 may impact the successful market penetration of infotainment packages and services. Advertising based on a user’s geolocation data or product preferences will be limited by privacy and data restrictions. Depending on the service, customers can create profiles with customised privacy and user settings. User profiles can be connected with in-vehicle infotainment systems and other third-party services within the vehicle. Additional consent requests and privacy requirements will be necessary for interconnected vehicle and user profile services. Third party services will also need to make explicit their intentions for the usage of their customer’s personal and geolocation data. The option for easy removal or deletion of profile data is also a requirement in EU regulation. For users not to be discouraged by the increased volume of data access and consent requests, third parties should consider uniform or simplified privacy settings that apply in-vehicle and out. 

If third parties intend on offering their services, the principals of privacy-by-design software development should be considered [67]. Developing a service with data privacy as a core requirement for the service will alleviate the effects of restrictions to data access, and its benefits are further detailed in Table 2. The change in the developing process can still be costly for the third party, as additional time and resources will be required to change their core software development model. A combination of privacy-by-design principals and engagement with OEMs on how their device processes/stores data, may significantly reduce the risks third parties experience when releasing their product.

### 5.3. Energy Reduction Schemes

Switching to an EV can significantly reduce GHG emission. In case study 4.3, we examined the limitations of inaccurate readings of battery state and health. Inaccurate readings of battery SOC introduces a significant level of range anxiety into the user of the vehicle. Attempts to further improve driver behavior models for fuel efficient driving or efficient battery usage in EVs are limited by access to private driver telematic data. This conflict of interest in data privacy and driver behavior modelling improvements will inevitably lead to inefficient driver feedback suggestions or battery health monitoring methods. Real-time data modelling may not be possible, but the usage of open naturalistic driving data sets can be considered as an intermediate alternative, which is further detailed in Table 2. This conflict of interest will need to be addressed by legislation, whereby real-time data access can be achieved for the purpose of fuel efficiency or driver behavior optimization suggestions.

### 5.4. Communication & Latency 

Third party and energy reduction schemes that are dependent on fast and reliable communication will be limited by the high latency and unreliable connections of DSRC and LTE-V2X networks. Services which require geolocation data for optimal route finding, smart intersections or other navigational third-party services are limited by the inability to send and receive traffic or environmental information reliably and efficiently. Limitations in these communication technologies are most notable in network congested areas such as intersections or traffic jams. Technologies which can alleviate the effects of poor networking speeds such as 5G, Adaptive Relay-Node Selection, edge caching, and vehicle fog technologies are currently in development and can be utilized for networking communications [73,74,75]. 5G can achieve 1ms latency for vehicle communications. Third party services and energy reduction schemes will have a short wait for efficient and reliable networking capabilities. These enhanced networking paradigms are summarized along with their benefits in Table 2.

## 6. Conclusions

In this review paper, we have demonstrated the significant technical and regulatory limitations of telematic devices. Telematic devices are limited in their data processing, memory storage and data transmission capabilities. Applications dependent on telematic devices will be limited or unviable due to these technical constraints. Our research has indicated that energy reduction schemes dependent on high-speed networking capabilities will be adversely affected by slow networking constraints of telematics devices. EVs which utilize telematic data for battery performance and health monitoring will be subject to inefficient estimations of SOC and, SOH due the technical constrains of these devices. Improvements in networking performance and enhanced computational capabilities of the device will alleviate these technical challenges, however our analysis has shown that advances in the computational capabilities of the device is subject to significant operational cost. 

The regulatory hurdles of restricted access to telematic data will invariably contribute significant operational inefficiencies in UBI and third-party services. Legislation requiring the installation of telematic devices in all new vehicles by 2024 will complicate the issue of data access and processing. Insurers and third parties will be restricted from accessing the telematic device directly and will require additional consent requests and alternative processes to access the telematic data. In this review, the process of in-host vehicle processing has been identified as a significant contributor to these operational costs. Additionally, access to geolocation data will be restricted, inhibiting insurers form accurately preventing and detecting fraud. Our analysis predicts that some insurers will inevitably be forced into an amalgamation of services to alleviate the substantial costs for the operation of UBI products. Insurers will additionally be required to consolidate, and lobby government and manufactures to standardize the minimum requirements of telematic devices. Governments and OEMs should also consider introducing uniform privacy preferences for users of the vehicle. The introduction of uniform privacy preferences should simplify and ease the burden or multiple consent requests for the user. We have also identified utilising the principals of privacy-by-design and blockchain-based data privacy technologies. We propose that these principals will facilitate in alleviating privacy concerns and may lead to additional regulation to support the introduction of these practices. 

Future research should consider how technical and regulatory limitations will affect connected autonomous vehicles, as these limitations may inhibit their operability. Additional research should consider regulation in other jurisdiction where comparisons could be drawn between the current and future perspectives of regulation on telematic devices. In this paper, we have provided a comprehensive analysis of the technical and regulatory limitations of telematic devices and proposed solutions aimed to alleviate the effects of these constraints on third parties, insurers, and the consumer. 

## Figures and Tables

**Table 1:** Summary of Technical/Regulatory Limitations.

Telematic Constraint	Application of Telematics	Limitation	Cost	Reference
Technical	Communication	DRSC	High Latency	[43,44]
		LTE-V2X	Unreliable	[45]
	Battery Monitoring System	Model Based (EM, ECM, EIM)	Inaccurate	[40]
		Machine Learning	Data requirements	[41]
	Storage	Current Memory 15MB	Increase to 25GB	[9,11]
Regulatory	Embedded Telematics	ePrivacy	Restricted Data Access	[47]
		GDPR	-	[48]
	User Consent Requirement	Ability to delete data	EU Requirement	[1,2]
		Profile Management		
	Host Vehicle Processing	Host processing	Computational Cost	[2] (p. 10)
		Third Party Processing	-	[1] (p. 16)
	Geolocation Restrictions	Limited Access	Driver Behavior	[51,52,53]
			Fraud Detection	[51]

**Table 2:** Summary of Suggestions and Benefits.

Application of Telematics	Limitation	Suggestion	Benefit	Cost
	Data Access and Privacy	Privacy-by-Design	Privacy and	−2% Revenue
		Blockchain Privacy	Data Access	Fine
	Geolocation	Homomorphic Encryption	Driver Behavior,	Deloitte
Insurance		Attribute-Based Encryption	Fraud Detection	
	Consolidation	OEM Consolidation	Standardization	~GBP 100 per Vehicle
		Third Party		>GBP 100 per Vehicle
Third Party Services	Data Access and Privacy	Privacy-by-Design	Privacy and Data Access	−2% Revenue
Energy Reduction Schemes	Battery Monitoring	Naturalistic Driving Data	Intermediate Alternative	Battery Life > 5 years
Communication and Latency	Latency and Reliability	5G, Edge Caching, Vehicle Fog	Increased Transmission Speed and Reliability	1 ms Latency

## Abbreviations

3GPP	Third Generation Partnership Project
ADAS	Advanced Driver Assistance Systems
C-V2X	Cellular-Vehicle-to-Everything
DSRC	Dedicated Short-Range Communication
EDPB	European Data Protection Board
ECM	Equivalent Circuit Model
EIM	Electrochemical Impedance Model
EM	Electrochemical Model
EV	Electric Vehicle
GDPR	General Data Protection Regulation
GHG	Green House Gas
ISP	Internet Service Provider
IWGDPT	International Working Group on Data Protection in Telecommunications
LTE-V2X	Long Term Evolution-Vehicle-to-Everything
OEM	Original Equipment Manufacturer
OJEU	Official Journal of the European Union
SOC	State of charge
SOH	State of Health
UBI	Usage Based Insurance
V2I	Vehicle-to-Infrastructure
V2V	Vehicle-to-Vehicle
V2X	Vehicle-to-Everything
